# Decrease in volume and density of foraminiferal shells with progressing ocean acidification

**DOI:** 10.1038/s41598-021-99427-1

**Published:** 2021-10-07

**Authors:** Azumi Kuroyanagi, Takahiro Irie, Shunichi Kinoshita, Hodaka Kawahata, Atsushi Suzuki, Hiroshi Nishi, Osamu Sasaki, Reishi Takashima, Kazuhiko Fujita

**Affiliations:** 1 Tohoku University Museum, Tohoku University, 6-3 Aramaki-aza Aoba, Aoba, Sendai, Miyagi 980-8578 Japan; 2 Atmosphere and Ocean Research Institute, The University of Tokyo, 5-1-5 Kashiwanoha, Kashiwa, Chiba 277-8564 Japan; 3 Department of Geology and Paleontology, National Museum of Nature and Science, 4-1-1 Amakubo, Tsukuba, Ibaraki 305-0005 Japan; 4 National Institute of Advanced Industrial Science and Technology (AIST), Higashi 1-1-1, Tsukuba, Ibaraki 305-8567 Japan; 5 Institute of Dinosaur Research, Fukui Prefectural University, Matsuo kakenjojima 4-1-1, Yoshida-gun Eiheijicho, Fukui, 910-1142 Japan; 6 Department of Physics and Earth Sciences, University of the Ryukyus, Senbaru 1, Nishihara, Okinawa 903-0213 Japan

**Keywords:** Palaeoecology, Environmental sciences, Ocean sciences, Planetary science

## Abstract

Rapid increases in anthropogenic atmospheric CO_2_ partial pressure have led to a decrease in the pH of seawater. Calcifying organisms generally respond negatively to ocean acidification. Foraminifera are one of the major carbonate producers in the ocean; however, whether calcification reduction by ocean acidification affects either foraminiferal shell volume or density, or both, has yet to be investigated. In this study, we cultured asexually reproducing specimens of *Amphisorus kudakajimensis,* a dinoflagellate endosymbiont-bearing large benthic foraminifera (LBF), under different pH conditions (pH 7.7–8.3, NBS scale). The results suggest that changes in seawater pH would affect not only the quantity (i.e., shell volume) but also the quality (i.e., shell density) of foraminiferal calcification. We proposed that pH and temperature affect these growth parameters differently because (1) they have differences in the contribution to the calcification process (e.g., Ca^2+^-ATPase and Ω) and (2) pH mainly affects calcification and temperature mainly affects photosynthesis. Our findings also suggest that, under the IPCC RCP8.5 scenario, both ocean acidification and warming will have a significant impact on reef foraminiferal carbonate production by the end of this century, even in the tropics.

## Introduction

Following the Industrial Revolution, anthropogenic atmospheric CO_2_ partial pressure (*p*CO_2_) increased rapidly, and the pH of seawater decreased. By 2100, atmospheric CO_2_ is predicted to increase to 420–1250 ppm^1^, with seawater pH decreasing to pH_total_ 7.6 (*p*CO_2_ = 1250 ppm; pH_NBS_ ~ 7.7) in the tropical Pacific^2^. Total dissolved CO_2_ concentration increases with *p*CO_2_, carbonate ions (CO_3_^2−^) decrease with decreasing pH, and the saturation state of calcium carbonate decreases. Calcifying organisms generally exhibit negative effects on survival, calcification, growth, and reproduction in response to ocean acidification^3^. Modern surface seawater is saturated with respect to calcium carbonate, including calcite, high-magnesian (high-Mg) calcite, and aragonite. However, it is expected that the seawater of the entire Southern Ocean south of 60° S and a part of the subarctic Pacific will become unsaturated with aragonite by 2100 (*p*CO_2_ = 563 or 788 ppm^4^).

Foraminifera are one of the major carbonate producers in the ocean, accounting for 23–56% of the total CaCO_3_ in the open ocean^5^. Foraminifera, both planktic and benthic, generally respond negatively to ocean acidification^6,7^. The reduction in the calcification rate in foraminifera has generally been estimated from shell weight and size, using the parameter of size-normalized shell weight^6^. Although several studies on shell density have been reported^8,9^, it remains unclear whether the change in shell weight in foraminifera of the same size reflects shell volume, shell density, or both, because it is difficult to measure their small shell volumes. The recently established micro X-ray computed tomography (microCT) technique enables direct and precise measurement of foraminiferal shell volume (i.e., density) with a resolution of less than 1 µm^10,11^. Therefore, microCT measurements can provide more precise information about foraminifera shell dimensions.

Large benthic foraminifera (LBFs) are predominantly distributed in warm and euphotic habitats and contribute to the organic and inorganic carbon production in coral reefs^12^. Most LBFs secrete high-Mg calcite (e.g., Miliolida; 8–18 mol% MgCO_3_)^13,14^, which have a higher solubility than aragonite^15^. *Amphisorus kudakajimensis*, formerly classified as *Marginopora kudakajimensis*, is a dinoflagellate endosymbiont-bearing LBF belonging to the Soritinae (high-Mg calcite). This species is common in shallow lagoon environments and accounts for 10% of the inorganic carbon production of protected lagoon communities^12,16^. Kinoshita et al.^11^ cultured the LBF *Sorites orbiculus* at different water temperatures and found that shell weight and volume increased with temperature, while density remained constant. However, the effect of pH on shell volume and density is not yet known. Although Kuroyanagi et al.^17^ cultured *A. kudakajimensis* under different pH conditions, they only examined the shell length and weight, and the effect of pH on shell volume and density remains unclear. Therefore, to examine the effect of ocean acidification on foraminiferal shell calcification and apply this to the estimation of past and future environmental changes in coral reefs, we cultured asexually reproducing specimens of *A. kudakajimensis* under different pH conditions and determined their shell volume and density using microCT.

## Results

**Figure 1:**
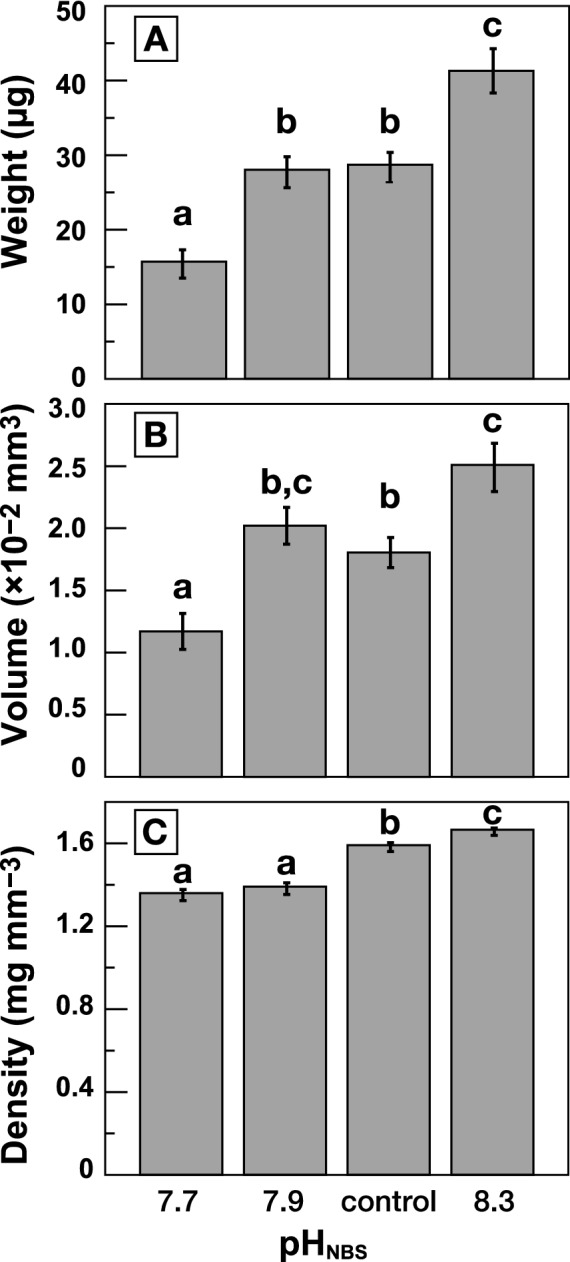
Mean shell (**A**) weight, (**B**) volume, and (**C**) density of *Amphisorus kudakajimensis* shells cultured under four pH conditions. Error bars indicate the standard errors of the corresponding mean values. Letters above the bars indicate significant differences according to Tukey’s HSD tests (α = 0.05) on shell weight^1/3^, volume^1/3^, and density. Tukey’s HSD tests were performed using JMP Pro statistical software (ver. 15.2.0 for Windows, SAS Institute Japan Ltd., Tokyo, Japan).

Our results demonstrate that shell weight, volume, and density are all positively associated with seawater pH (Table 1 and Fig. 1). The null hypothesis that the mean weight is equal across the four pH conditions was rejected (*n* = 111, *df* = 3, *F* = 24.2, *P* < 0.0001). Similarly, an ANOVA reported significant differences between the mean shell volume of the four pH treatments (*n* = 111, *df* = 3, *F* = 15.1, *P* < 0.0001) and between the mean shell density (*n* = 111, *df* = 3, *F* = 41.5, P < 0.0001). Tukey's HSD test reveals statistically significant difference between pH 7.7 and control and pH 8.3 in all shell volume, weight, and density. However, the pH 7.9 condition showed different results for each parameter: there was no statistically significant difference in shell density compared to the pH 7.7 condition, in shell weight compared to the control condition, or in volume compared to either the control or pH 8.3 conditions (Fig. 1).

**Table 1:** Shell weight, volume, and density of cultured *Amphisorus kudakajimensis* under four different pH conditions.

Treatments (pH of cultured water)	n	Shell weight (µg)					Shell volume (×10^−2^ mm^3^)					Shell density (mg mm^−3^)				
		Mean	Min.	Max.	Std. Dev.	Std. Error	Mean	Min.	Max.	Std. Dev.	Std. Error	Mean	Min.	Max.	Std. Dev.	Std. Error
pH 7.7	23	15.7	4.0	35.1	9.0	1.9	1.17	0.32	2.55	0.69	0.14	1.36	1.17	1.63	0.13	0.03
pH 7.9	17	28.0	13.1	43.9	8.5	2.1	2.02	0.84	3.34	0.63	0.15	1.39	1.14	1.57	0.12	0.03
Control	36	28.7	13.0	69.0	12.0	2.0	1.80	0.84	4.20	0.73	0.12	1.59	1.13	2.00	0.13	0.02
pH 8.3	35	41.3	18.1	90.3	17.5	3.0	2.51	1.09	5.47	1.15	0.19	1.67	1.36	1.83	0.10	0.02

Both mean shell weight and density increase with pH, and they range 15.7–41.3 µg and 1.36–1.67 mg mm^−3^, respectively (Table 1 and Fig. 1). Mean volume at pH 8.3 (2.51 × 10^−2^ mm^3^) is more than twice as large as that at pH 7.7 (1.17 × 10^−2^ mm^3^) even though it is slightly higher at pH 7.9 (2.02 × 10^−2^ mm^3^) than at control (1.80 × 10^−2^ mm^3^). When deformed individuals are removed from the data, these trends are maintained at pH 7.7 (*n* = 18), pH 7.9 (*n* = 14), control (*n* = 35), and pH 8.3 (*n* = 33) (see Supplementary Table S3).

## Discussion

**Figure 2:**
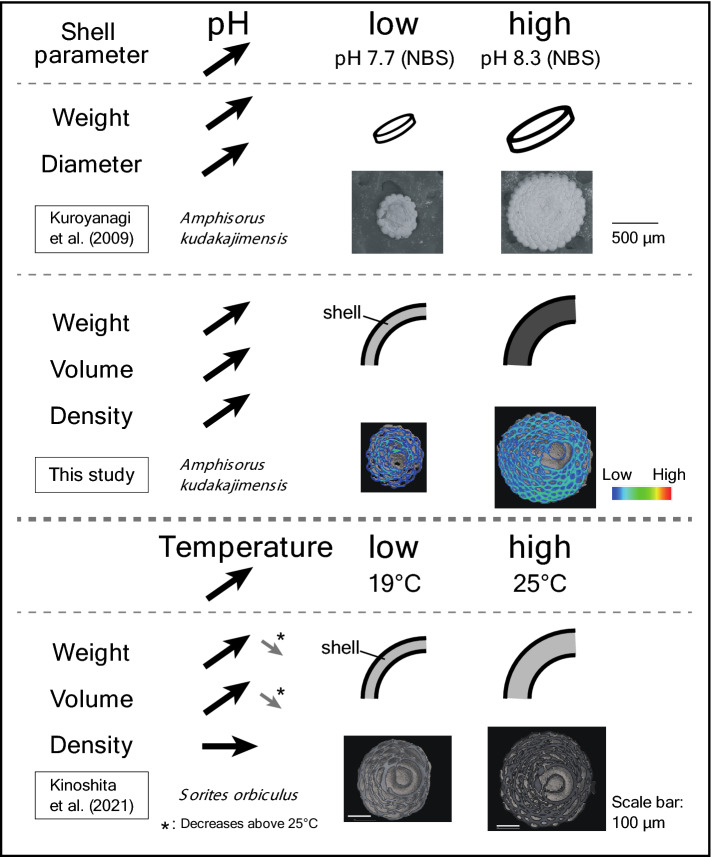
The relationship between shell parameters (weight, diameter, volume, and density) of cultured Soritinae (*Amphisorus kudakajimensis* and *Sorites orbiculus*) and pH and water temperature. MicroCT photo was created using the 3D image processing software Molcer Plus (ver. 1.6, White Rabbit Corp., Tokyo, Japan). The color of the MicroCT photo (#37 and #85 samples) represents the X-ray absorption coefficient (i.e., warm colors mean relatively high density).

Ocean acidification has resulted a decrease in the saturation state of calcium carbonate (Ω), and foraminifera (both planktic and benthic) have been found to respond negatively to ocean acidification^2,6,7^. The decrease in the calcification rate of foraminifera has generally been inferred from the weight and size of the shell (i.e., size-normalized shell weight, shell area density^18,19^). Shell density in relation to ocean acidification has also been reported in several studies^8,9^, however, until now, it has been unclear whether the change in shell weight is a reflection of changes in shell volume, shell density, or both, due to the difficulty of measuring the volume of tiny shells. In this study, we found that both the volume and density of the foraminiferal shells decreased with decreasing pH, based on volume measurements using microCT (Fig. 2). It suggests that changes in pH affect not only the quantity (i.e., shell volume) but also the quality (i.e., shell density) of foraminiferal calcification.

Weight, volume, and density of the *A. kudakajimensis* shells all decreased with decreasing pH in our study (Fig. 1). Kinoshita et al.^11^ cultured clonal juvenile individuals of *Sorites orbiculus*, which belong to the same subfamily as *Amphisorus*, under six different temperatures (19–29 °C). MicroCT measurements showed that the shell weight and volume increased with temperature up to ~ 25 °C, whereas the density remained unchanged from pre-culturing (Fig. 2). Together, these findings show that pH affects both shell volume and density, while temperature affects shell volume only, at least within the tested range (Fig. 2). Similarly, in corals, ocean acidification affected skeletal density (lateral thickening), rather than extension (upward growth), by a fundamentally different process^20^. It suggests that either pH and temperature modulate different calcification mechanisms and/or that they regulate the same process but with different degrees of influence. The culturing studies of *Marginopora vertebralis,* also belonging to the same subfamily *Amphisorus*, was reported in which *p*CO_2_ and temperature (above 28 °C) were varied^21,22^. However, they had used surface area or buoyant weight as the growth rate, thus we could not confirm the consistency of their results with ours. Therefore, further investigation of other shell parameters (e.g., shell volume and density) would provide valuable information. The rate-limiting step for calcification in *Amphisorus hemprichii* should be diffusion (i.e., CO_3_^2^ concentration), while Ca uptake occurs by both diffusion and enzyme-mediated uptake^23^. The optimum pH for Ca^2+^-ATPase (Ca^2+^-pump) and alkaline phosphatase, which may be an important enzyme in the process of calcification^24^, was pH_total_ 8.0 and 10.2 (pH_NBS_ ~ 8.15 and ~ 10.35), respectively^25^. However, the activity of Ca^2+^-ATPase also increased with temperature, despite the lower rate of activity compared to the increase in pH^25^. Carbonate saturation state also increased with both pH and water temperature, though the rate of increase was higher for pH than for temperature (between 7.7 and 8.3 for pH_NBS_ and between 15 °C and 28 °C for temperature)^2^. Thus, pH contributes significantly to the activity of the enzymes related to calcification and changes in saturation state, while temperature has a relatively small effect. Ter Kuile et al.^23^ observed that the calcification rate of *A. hemprichii* increased with increases in the external concentration of pH (pH 7.6–9.5) and inorganic carbon (0.1–3.8 mM), whereas the photosynthetic rate showed Hill-Whittingham type kinetics and maintained relatively constant around that of normal seawater conditions. On the other hand, the culturing results of *M. vertebralis* showed that temperature had more impact on photosynthesis than changes in ambient CO_2_^22^. Therefore, (1) the different contribution of pH and temperature to the calcification process, and (2) pH mainly affects calcification, and water temperature mainly affects photosynthesis may be the reason for the different effects of pH and temperature on shell growth. Prazeres et al.^8^ cultured adult (i.e., grown in the control condition) *M. vertebralis* under lowered pH, and found no effect on growth rate (surface area and buoyant weight), skeletal density, or Ca-ATPase activity. Similarly, an increase in growth rate (surface area), even under high *p*CO_2_ (1169 and 1662 µatm), has been reported from the same adult *M. vertebralis* culturing^26^. It suggests the biological response to ocean acidification would differ depending on the growth stage, with the juvenile being more affected.

Foraminiferal shells can record their habitat conditions, thus the shell information including volume and density can be useful as an environmental proxy. For example, the δ^18^O and Mg/Ca ratio of the shell are well known as proxies for water temperature^27^. Numerous studies have discussed the potential of planktic shell information (e.g., shell weight, size, size-/area-normalized shell weight) as a proxy for environmental parameters; ambient seawater [CO_3_^2−^]^18,28–31^, *p*CO_2_^18,32,33^, temperature^33,34^ etc. In addition, several other environmental parameters, such as nutrients, salinity, and marine algae have also been reported to have affected shell calcification^35–39^. Osborne et al.^19^ examined the shell area density (area-normalized shell weight) from sediment trap samples and proposed that the intensity of calcification is primarily controlled by [CO_3_^2−^], whereas temperature influences shell size. Since *p*CO_2_ generally increases with the increase in water temperature, it is difficult to examine the effects of these two parameters separately in field studies. Our culturing results indicate that pH affects both shell volume and density of LBF, while water temperature affects only the shell volume. Therefore, the records of shell volume and density can provide information on changes in pH and water temperature, at least within the ranges of pH and temperature examined (Fig. 2). Unfortunately, the calcification mechanism is known to differ between *Sorites* LBF (imperforate; porcelaneous or miliolid) and some benthic and planktic foraminifera (perforate; hyaline)^23,40^. Indeed, LBFs between different calcification types and symbionts (dinoflagellate/diatom) also exhibited different responses to acidification^7,8,14,21,26^. Therefore, more detailed studies of hyaline species such as planktic foraminifera will be required in the future. The boron isotopes (δ^11^B) of planktic foraminiferal shell reflects the seawater pH and has been used as a proxy for pH in reconstructing past environments^41–43^. If the shell information from planktic foraminifera is also found to reflect changes in the marine environment, it could potentially be a valuable proxy for estimating past seawater pH, in addition to δ^11^B.

Based upon the Intergovernmental Panel on Climate Change (IPCC) RCP 2.6–8.5 scenarios^1^, it is predicted that, by 2100, atmospheric CO_2_ will increase to 420–1250 ppm and global mean temperature will rise by ~ 1.0 to 3.7 °C (range 0.3–4.8 °C^1^). If this happens, seawater pH will decrease to as low as 7.6 (*p*CO_2_ = 1250 ppm; pH_NBS_ ~ 7.7) in the tropical Pacific^2^. Simultaneous increases in seawater temperature will also make marine organisms more susceptible to ocean acidification^44^, thus a serious decline in coral reef carbonate production is predicted^45,46^. In our study, the results of the comparisons between the pH 7.9 condition and the other conditions were inconsistent across the different parameters, which could potentially be explained by the presence of thresholds or optimum values around pH 7.9; for example, the optimal pH for Ca^2+^-ATPase is found between pH 7.9 and control^25^. On the other hand, the measures of shell volume, weight, and density in the pH 7.7 condition (*p*CO_2_ =  ~ 1250 ppm) were all statistically significantly different from those in the control and pH 8.3 conditions (Fig. 1). Temperature also will increase with *p*CO_2_, and all LBFs showed reduced calcification and photosymbiont health under long-term high temperatures, referred to as 2100 in the RCP8.5 scenario^7,21,22^. The shell weight of *Sorites* LBF decreased by 45.3% when pH decreased from control to pH 7.7 (Table 1), and by 28.3% when water temperature increased from 25 to 29 °C^11^. Under the RCP8.5 scenario, ocean acidification and warming will have a significant impact on reef foraminiferal carbonate production (43 million tons of current carbonate production per year^47^) by the end of this century, even in the tropics, which is considered to be the least sensitive region in the global oceans because of its higher Ω that results from higher temperatures in that region.

## Materials and methods

### Sampling and culturing methods

Mature, living individuals of *A. kudakajimensis* (Gudmundsson, 1994) were collected from Okinawa, Japan (26° 39′ N, 127° 51′ E), in early May 2008 and underwent asexual reproduction (multiple fission) in the laboratory (Ocean Research Institute, University of Tokyo). Three-day-old individuals from an asexually produced brood were randomly allocated into four glass jars. Each jar contained 110 ml of filtered natural seawater with different pH levels. The control medium contained seawater at pH ~ 8.2 on the NBS scale; in the experimental conditions, the pH was adjusted to pH 7.7, 7.9, or 8.3 with the addition of 0.1 N HCl or NaOH, respectively. The jars were kept in a thermostatic bath at 25 °C, under high-intensity discharge lights with a photosynthetic photon flux density of 190 µmol m^−2^ s^−1^ and a 12:12 h light:dark cycle (Supercool 115, 150 W, Okamura, Chiba, Japan). We replaced the culture medium every week (7–9 days) and the pH was measured before and after the replacement. Although the pH of the culture medium may have changed slightly due to metabolic activity (e.g., respiration, calcification, and photosynthesis) of the foraminifera and their algal symbionts, we were successful at keeping the average pH of the medium within 0.03–0.24 pH units of the nominal values throughout the experimental period. The calcite saturation state (Ω_calcite_) were calculated using CO2SYS^48^, and these values were 1.8, 2.8, 6.2, and 5.1 at pH levels of 7.7, 7.9, 8.3, and control, respectively. More details about the sampling area and culturing methodology are described in Kuroyanagi et al.^17^.

In total, we cultured 122 *A. kudakajimensis* individuals at the four different pH values: pH 7.7 (*n* = 23), 7.9 (*n* = 18), 8.3 (*n* = 35), and pH ~ 8.2 (control condition, *n* = 36). After 6 and 8 weeks of culture, we measured the maximum shell diameter of 15–19 randomly selected individuals from each condition to examine their growth rate (see Ref.^17^). After 10 weeks (71 days) of culture, we measured the dry shell weight, shell volume, and maximum shell length (diameter) of all cultured specimens. Each shell was weighed separately using a microbalance (XP2U, Mettler-Toledo International Inc., Tokyo, Japan), which can measure weights down to 0.1 µg with a precision of 0.15 µg. Organic matter makes up ~ 3% of the dry shell weight^12^; thus, any differences between conditions will be negligible.

### MicroCT measurements

All cultured individuals were investigated at high-resolution using the X-ray microCT scanner at the Tohoku University Museum (resolution 1.652 µm/pixel, source voltage 100 kV, source current 40 µA, rotation step 0.18°; ScanXmate-D160TSS105/11000, Comscantecno Co. Ltd., Kanagawa, Japan). In order to verify the precision and repeatability of the LBF shell volume measurements, one foraminiferal individual cultured in the control medium was used as a standard sample for each measurement (Supplementary Tables S1 and S2). For each measurement, eight specimens and one standard specimen were fixed on solid Jefline glue (SEC Corp., Hakodate, Japan) with tragacanth gum and placed on the sample stage. 3D reconstructions of the foraminiferal shells were carried out using ConeCTexpress (White Rabbit Corp., Tokyo, Japan). The whole shell volume and diameter (i.e., including the pre-culturing shell) were measured using the 3D imaging software Molcer Plus (ver. 1.6, White Rabbit Corp., Tokyo, Japan). Shell volume was calculated as the sum of the 3D voxels, and the gray scale range was decided by the histogram of each shell. Repeated measurements (n = 15) showed that the shell volume of the standard sample ranged from 1.44 to 1.58 × 10^−2^ mm^3^ (mean = 1.49 × 10^−2^, *SD* = 0.03 × 10^−2^, *SE* = 0.01 × 10^−2^ mm^3^; Supplementary Table S2); shell volume was therefore measured to three significant figures in this study.

### Statistical analysis

We analyzed shell volume, weight, and density against the null hypothesis that their arithmetic means were equal across the four pH treatments. Next, we conducted pairwise mean comparisons between each of the treatments. The statistical tests used in this study perform best when the population distributions being compared are normal and homoscedastic. We therefore compared the results of the Shapiro–Wilk test of normality and Bartlett's test for homogeneity of variances from the untransformed, square-root transformed, cubic-root transformed, and arc-tangent transformed data for each of the three variables^49,50^. Consequently, we concluded that shell volume^1/3^, weight^1/3^, and density should be subject to the following statistical analyses. These three variables were each analyzed separately using ANOVA models with seawater pH as a fixed-effect. Between-treatment pairwise comparisons were conducted with Tukey's HSD tests. All analyses were performed using JMP Pro statistical software (ver. 15.2.0 for Windows, SAS Institute Japan Ltd., Tokyo, Japan).

## Supplementary Information

Supplementary Tables.
