# Supplementary material for: Decrease in volume and density of foraminiferal shells with progressing ocean acidification
Source: Sci Rep. 2021 Oct 7;11:19988. doi: 10.1038/s41598-021-99427-1 (PMC8497592; doi:10.1038/s41598-021-99427-1)
Supplement: Supplementary file 1 — Supplementary Tables. [file 41598_2021_99427_MOESM1_ESM.pdf]

Supplementary Information

Decrease in volume and density of foraminiferal shells with progressing ocean acidification

Azumi Kuroyanagi, Takahiro Irie, Shunichi Kinoshita, Hodaka Kawahata, Atsushi Suzuki, Hiroshi Nishi, Osamu Sasaki, Reishi Takashima, Kazuhiko Fujita

Table S1. Shell weight, volume, and density of cultured *Amphisorus kudakajimensis* under four different pH conditions.

Table S2. The MicroCT results of repeated volume measurements of *Amphisorus kudakajimensis* (control treatment) used as a standard sample.

Table S3. Shell weight, volume, and density of cultured *Amphisorus kudakajimensis* under four different pH conditions, after excluding deformed individuals.

Table S1
Shell weight, volume, and density of cultured *Amphisorus kudakajimensis* under four different pH conditions.

Treatments	n	sample #	weight (μg)	shell volume ($\times 10^{-2}$ mm^3)	density (mg mm^{-3})	sample name	note
**pH 7.7**	23	1	8.9	0.70	1.26	36	
		2	9.3	0.80	1.17	37	
		3	12.2	0.77	1.58	38	
		4	17.0	1.36	1.25	39	
		5	9.1	0.75	1.21	40	
		6	28.9	2.28	1.27	41	
		7	16.3	1.11	1.47	42	
		8	13.7	0.87	1.57	43	
		9	17.7	1.25	1.42	44	
		10	15.3	1.05	1.45	45	Deformed individual
		11	8.0	0.59	1.36	46	
		12	18.9	1.33	1.42	47	
		13	5.9	0.42	1.42	48	Deformed individual
		14	6.8	0.42	1.63	49	Deformed individual
		15	4.0	0.32	1.26	50	
		16	9.4	0.75	1.26	51	
		17	32.2	2.55	1.26	52	
		18	11.9	0.92	1.30	53	Deformed individual
		19	35.1	2.48	1.42	54	
		20	21.0	1.78	1.18	55	
		21	28.2	2.07	1.36	56	
		22	5.8	0.41	1.42	57	
		23	26.0	1.96	1.33	58	
**pH 7.9**	17*	1	27.7	1.90	1.46	59	
		2	30.1	2.34	1.29	60	
		3	28.0	1.88	1.49	61	Deformed individual
		4	13.1	0.84	1.57	62	
		5	35.5	2.50	1.42	63	
		6	32.1	2.23	1.44	64	
		7	43.9	3.34	1.32	65	
		8	13.5	1.04	1.30	66	
		9	25.5	1.82	1.40	67	
		10	33.2	2.32	1.43	68	
		11	26.4	1.81	1.46	69	
		12**	ND	1.85	ND	70	Deformed individual
		13	21.6	1.85	1.17	71	
		14	28.5	1.89	1.51	72	Deformed individual
		15	16.6	1.19	1.40	73	
		16	29.1	1.97	1.47	74	
		17	42.0	3.00	1.40	75	Deformed individual
		18	29.6	2.60	1.14	76	
**Control**	36	1***	23.3	1.51	1.54	standard	
		2	21.0	1.35	1.56	1	
		3	18.4	1.30	1.42	2	
		4	13.0	0.84	1.55	3	
		5	19.1	1.29	1.48	4	
		6	30.7	1.86	1.65	5	
		7	29.1	1.46	2.00	6	
		8	25.0	1.45	1.72	7	
		9	29.0	2.56	1.13	8	
		10	26.1	1.55	1.68	9	
		11	24.4	1.55	1.58	10	
		12	23.3	1.42	1.65	11	
		13	28.1	1.77	1.59	12	
		14	15.6	0.99	1.57	13	
		15	16.0	1.09	1.47	14	
		16	17.1	1.02	1.67	15	

		17	29.9	1.87	1.60	16	
		18	24.4	1.52	1.61	17	
		19	69.0	4.20	1.64	18	
		20	31.0	1.87	1.66	19	
		21	18.7	1.28	1.47	20	
		22	23.5	1.43	1.65	21	
		23	51.5	3.24	1.59	22	
		24	46.6	2.81	1.66	23	
		25	28.1	1.78	1.57	24	
		26	38.8	2.49	1.56	25	
		27	19.1	1.14	1.67	26	
		28	45.3	2.84	1.60	27	
		29	33.8	2.26	1.50	28	
		30	33.4	2.21	1.51	29	
		31	17.8	1.09	1.63	30	
		32	19.6	1.24	1.58	31	
		33	30.7	2.01	1.53	32	
		34	39.9	2.51	1.59	33	
		35	22.8	1.38	1.65	34	
		36	50.1	2.79	1.80	35	Deformed individual
**pH 8.3**	35	1	33.9	2.09	1.62	77	
		2	72.9	4.62	1.58	78	
		3	24.7	1.40	1.76	79	
		4	57.5	4.24	1.36	80	
		5	66.7	4.28	1.56	81	
		6	42.3	2.52	1.68	82	
		7	39.9	2.54	1.57	83	
		8	23.1	1.30	1.78	84	
		9	46.0	2.64	1.74	85	
		10	34.4	2.00	1.72	86	
		11	18.1	1.09	1.66	87	
		12	90.3	5.47	1.65	88	
		13	77.6	5.14	1.51	89	
		14	28.6	1.79	1.60	90	
		15	40.0	2.20	1.82	91	Deformed individual
		16	28.1	1.80	1.56	92	
		17	49.8	3.08	1.62	93	
		18	68.4	4.29	1.60	94	
		19	45.2	2.63	1.72	95	
		20	24.1	1.64	1.47	96	
		21	39.2	2.41	1.63	97	
		22	29.3	1.77	1.65	98	
		23	30.7	1.72	1.79	99	Deformed individual
		24	29.9	1.74	1.72	100	
		25	39.6	2.37	1.67	101	
		26	23.7	1.35	1.76	102	
		27	37.3	2.18	1.71	103	
		28	18.1	1.12	1.61	104	
		29	35.4	1.93	1.83	105	
		30	59.7	3.39	1.76	106	
		31	42.1	2.40	1.75	107	
		32	48.1	2.75	1.75	108	
		33	20.6	1.22	1.69	109	
		34	39.6	2.27	1.74	110	
		35	40.4	2.41	1.68	111	

*: #12 is not included

**: Lost after MicroCT and before weight measurement.

***: Repeated measurements with MicroCT as a standard sample (see Table S2).

Table S2

The MicroCT results of repeated volume measurements of *Amphisorus kudakajimensis* (control treatment) used as a standard sample.

Treatments	sample #	shell volume ($\times 10^{-2}$ mm^3)	sample name
**Control**	Std-1	1.47	STD77_1
	Std-2	1.48	STD77_2
	Std-3	1.44	STD77_3
	Std-4	1.49	STD79_1
	Std-5	1.49	STD79_2
	Std-6	1.52	STD79_3
	Std-7	1.54	STD83_1
	Std-8	1.58	STD83_2
	Std-9	1.48	STD83_3
	Std-10	1.45	STD83_4
	Std-11	1.48	STD_c1
	Std-12	1.48	STD_c2
	Std-13	1.51	STD_c3
	Std-14	1.47	STD_c4
	Std-15	1.49	STD_NBS

Table S3
Shell weight, volume, and density of cultured *Amphisorus kudakajimensis* under four different pH conditions, after excluding deformed individuals.

Treatments (pH of cultured water)	n	Shell weight (μg)					Shell volume ($\times 10^{-2}$ mm^3)					Shell density (mg mm^{-3})				
		Mean	Min.	Max.	Std. Dev.	Std. Error	Mean	Min.	Max.	Std. Dev.	Std. Error	Mean	Min.	Max.	Std. Dev.	Std. Error
pH 7.7	23	17.2	4.0	35.1	9.6	2.3	1.30	0.32	2.55	0.72	0.17	1.33	1.17	1.57	0.11	0.03
pH 7.9	17	27.0	13.1	43.9	8.6	2.3	1.98	0.84	3.34	0.66	0.18	1.37	1.14	1.57	0.12	0.03
control	36	28.1	13.0	69.0	11.6	2.0	1.78	0.84	4.20	0.72	0.12	1.59	1.13	2.00	0.13	0.02
pH 8.3	35	41.7	18.1	90.3	18.0	3.1	2.54	1.09	5.47	1.17	0.20	1.66	1.36	1.83	0.10	0.02
